# S05-2: National Strength in Old Age Program: 20 years of committed municipalities and intersectoral collaboration

**DOI:** 10.1093/eurpub/ckae114.219

**Published:** 2024-09-26

**Authors:** Heli Starck, Pirjo Kalmari, Saila Hänninen, Katja Borodulin

**Affiliations:** Age Institute; Age Institute; Age Institute; Age Institute

## Abstract

**Purpose:**

To prepare for increasing numbers of older persons, a National Program to promote functional capacity among older persons aged 75+ years was launched.

**Development:**

The Strength in Old Age Program (on-going since 2004) in Finland aims to launch health-enhancing exercise for independently living older adults with decreased functional capacity. Age Institute coordinates the Program and mentors the municipalities, each participating for a three-year-period. The Program is primarily funded by Ministry of Social Affairs and Health.

**Implementation:**

Participating municipalities implement good practices in exercise counseling, strength and balance exercise and outdoor exercise. They commit to implementation through intersectoral collaboration without extra funding. The sectors include eg. social, health care, sports services, and NGOs. Local cross-sectoral work is supported by the mentoring of Age Institute including counseling, continuing education, materials, network events, development tools, and collection of follow-up data.

**Evaluation:**

109 municipalities out of 297 have participated in the Program. Results from the municipalities state that 79% perceived receiving high or very high benefits from participation in the Program and 89% were committed to continue the good practices after the 3-year mentoring phase. They were most satisfied with tailored counseling, free continuing education, and materials. They also met challenges, e.g. high turnover of workers and lack of support from decision-makers. The Program develops solutions to local challenges by learning from others and using the good practices.

The amount of exercise groups has increased in most municipalities. A survey of exercise group participants has been made in three waves (2015-2023). The participants perceived mostly good or strong benefits from their participation in the groups. Physical functioning test results show that majority of the participants improved their strength and balance.

**Dissemination:**

A systematic dissemination has been carried out by Age Institute. Scale-up is limited to availability of resources. Until 2023, individual municipalities applied to the 3-year-Program. In future, the Program will focus the dissemination to Wellbeing Services Counties, after the social and health care reform.

**Conclusions:**

The Program has shown substantial results in all participating municipalities, where key elements include commitment of the municipalities, support from Age Institute and participation of older persons.

